# Repurposing CPAP machines as stripped-down ventilators

**DOI:** 10.1038/s41598-021-91673-7

**Published:** 2021-06-09

**Authors:** J. Nguyen, K. Kesper, G. Kräling, C. Birk, P. Mross, N. Hofeditz, J. Höchst, P. Lampe, A. Penning, B. Leutenecker-Twelsiek, C. Schindler, H. Buchenauer, D. Geisel, C. Sommer, R. Henning, P. Wallot, T. Wiesmann, B. Beutel, G. Schneider, E. Castro-Camus, M. Koch

**Affiliations:** 1 Faculty of Physics and Material Sciences Centre, Philipps-Universität Marburg, Marburg, Germany; 2 Department of Pneumology, Philipps-Universität Marburg, Marburg, Germany; 3 Department of Medical Technology, Universitätsklinikum Gießen und Marburg, Marburg, Germany; 4 Department of Neurology, Universitätsklinikum Gießen und Marburg, Marburg, Germany; 5 Faculty of Mathematics and Computer Science, Philipps-Universität Marburg, Marburg, Germany; 6 TRUMPF, Ditzingen, Germany; 7 Schneider GmbH & Co KG, Fronhausen, Germany; 8 Department of Anaesthesiology & Intensive Care Medicine, Universitätsklinikum Gießen und Marburg, Marburg, Germany; 9 Department of Medicine, Pulmonary and Critical Care Medicine, Universitätsklinikum Gießen und Marburg, Member of the German Centre for Lung Research (DZL), Marburg, Germany; 10 Centro de Investigaciones en Optica, A.C., Loma del Bosque 115, Lomas del Campestre, 37150 Leon, Guanajuato Mexico

**Keywords:** Engineering, Respiratory tract diseases, Respiratory distress syndrome

## Abstract

The worldwide shortage of medical-grade ventilators is a well-known issue, that has become one of the central topics during the COVID-19 pandemic. Given that these machines are expensive and have long lead times, one approach is to vacate them for patients in critical conditions while patients with mild to moderate symptoms are treated with stripped-down ventilators. We propose a mass-producible solution that can create such ventilators with minimum effort. The central part is a module that can be attached to CPAP machines and repurpose them as low-pressure ventilators. Here, we describe the concept and first measurements which underline the potential of our solution. Our approach may serve as a starting point for open-access ventilator technologies.

## Introduction

The COVID-19 pandemic plunged many countries, such as Italy, Spain and the United States, into a health crisis due to the sudden demand for medical facilities, staff and equipment^1,2^. Since the global vaccination process is still ongoing, treatments revolve around symptom control and life-sustaining measurements. Patients with COVID-19-related respiratory failures often require prolonged mechanical ventilation^3–5^. Such a treatment, however, cannot be provided due to a long-term worldwide issue: the shortage of medical-grade ventilators^6,7^. These costly devices have long lead times because of their complexity. Consequently, this shortage may continue to be one of few central issues during subsequent waves of the disease.

In response to the urgent need for ventilators, many designs were proposed to serve as life-sustaining measurements or to vacate medical-grade ventilators for patients in critical conditions^8^. The lead time for each proposed design depends on the range of functionalities, cost and, more importantly, the available production facilities. One prominent approach focuses on machines that automatically compress and decompress bag valve masks^9–13^. Other groups pursue ventilator solutions that can be built from scratch with the expertise and the facilities from different industrial branches^14–16^.

In the presence of these developments, we propose a mass-producible solution that is built around continuous positive airway pressure (CPAP) machines. These home-level ventilators are commonly used to treat sleep apnea worldwide. Nowadays, many machines sit unused in households^17–19^ and thus, there are potentially millions of CPAP machines around the globe that can be repurposed as stripped-down ventilators.

CPAP machines generate and maintain a constant positive airway pressure to the patient. To cover the fundamental functions for mechanical ventilation, however, we need to:

- provide two well-defined airway pressure levels, i.e. the peak inspiratory pressure (P_IP_) and the positive end-expiratory pressure (PEEP), to assist or replace the patient’s breathing during the inhalation and exhalation phase,
- set the respiratory rate in units of breaths per minute (bpm),
- set the I:E ratio, which is the ratio of the inhalation and the exhalation time, and
- allow patients to trigger and to cycle inhalation.

Therefore, ventilation by means of CPAP machines requires a modification that regulates the constant pressure with time. We developed a module, referred to as CARL (for **C**PAP **A**pparatus **R**espiratory **L**ife support), that can be attached to any CPAP machine and instantaneously repurpose it as a basic ventilator with minimum effort. We believe that our proposed solution can be used to treat patients with mild symptoms that do not require high-level pressures provided by medical-grade ventilators or to wean off recovering patients from the ventilator.

We would like to clarify that the ventilator presented in this report:

- has only been tested with electric and mechanic equipment,
- is subject to pre-/clinical trials and approval, and
- has yet to be examined and approved by a regulatory health institution.

Therefore, at this stage of development, we strongly discourage the use of our device on humans. Our module should only be reproduced for the purpose of further development and investigation.

## Results

CARL is an electronic device that is placed in the ventilation pathway between the CPAP machine and the patient. It uses a microcontroller-activated valve to generate two airway pressure levels from the constant pressure provided by the CPAP machine. The computer assures that the periodic alteration between both pressure levels corresponds to a set respiratory rate and I:E ratio. CARL uses pressure sensors to monitor the airway pressure, the flow, and the ventilation volume. That way, users can easily adjust the ventilator settings to the needs of the patient which is comparable to adjustments with medical-grade ventilators.

In the following, we present measurements of the airway pressure, flow, and ventilation volume (referred to as ventilation curves) for different ventilation parameters. As a representative patient model, we use an artificial lung (details in ‘Methods’).

### Pressure-related ventilation settings

**Figure 1:**
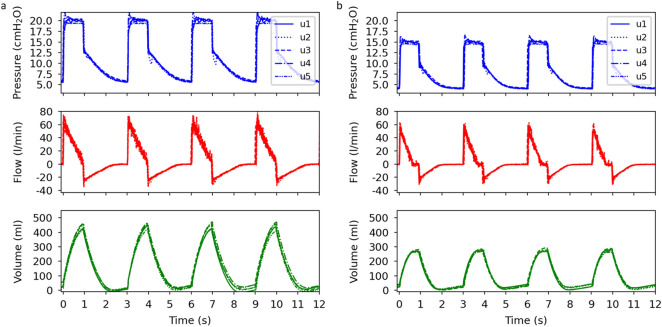
Plots of the airway pressure, flow, ventilation volume as a function of time for the P_IP_ values (**a**) 20 cmH_2_O and (**b**) 15 cmH_2_O. Each curve corresponds to a ventilator with a different CPAP machine model (referred to as u1, u2, u3, u4 and u5). All measurements are performed on an artificial lung. Respiratory rate: 20 bpm, I:E ratio: 1:2.

In our ventilator solution, the pressure is built up and maintained by a CPAP machine. Since each machine has its own feedback control system, the ventilation performance may vary with the machine model. To shed light on this, we test CARL with five different CPAP machine models while setting the P_IP_ value, respiratory rate, and I:E ratio to 20 cmH_2_O, 20 bpm and 1:2, respectively. The ventilation curves for each ventilator are presented in Fig. 1a. A comparison of the curve profiles between the ventilators reveals minor differences. This shows that each selected machine can quickly adapt to sudden pressure changes that are caused by CARL. We also see that the pressure in the plateau region depends on the selected CPAP machine: for four out of five machines, the plateau pressure stabilises around the set P_IP_ value, while for machine u5 the plateau pressure approaches 19.4 cmH_2_O. The latter observation is due to a less powerful motor compared to the other machines. On the other hand, each ventilator can keep the PEEP value above 5 cmH_2_O which aligns with ventilation strategies for COVID-19-patients where a minimum airway pressure of at least 5 cmH_2_O must be maintained during the exhalation phase to stabilise the alveoli^5,20–23^.

To investigate the impact of different P_IP_ values, we repeated the same measurements but changed the P_IP_ value to 15 cmH_2_O. The ventilation curves are presented in Fig. 1b. Here, we make the same observation as before with the difference that the plateau pressure and the PEEP value are reduced. Note, that for this case the PEEP value is below 5 cmH2O which could be increased by adjusting CARL’s design.

Both measurements demonstrate that our CARL-based ventilator can generate two pressure levels to produce ventilation patterns typical for mechanical ventilation^24^. The acquired ventilation curves are comparable to the ones produced by already published mechanical ventilator solutions^9–16^ that vary with different sets of functionalities.

### Time-related ventilation settings

Time-related ventilation settings, i.e. the respiratory rate and the I:E ratio, are adjusted on CARL. In the following, we only consider the ventilation data taken with the machine u1 (prisma SOFT, Löwenstein Medical GmbH & Co. KG), since Fig. 1a,b show that the ventilation performances between the CPAP machine models are similar.

**Figure 2:**
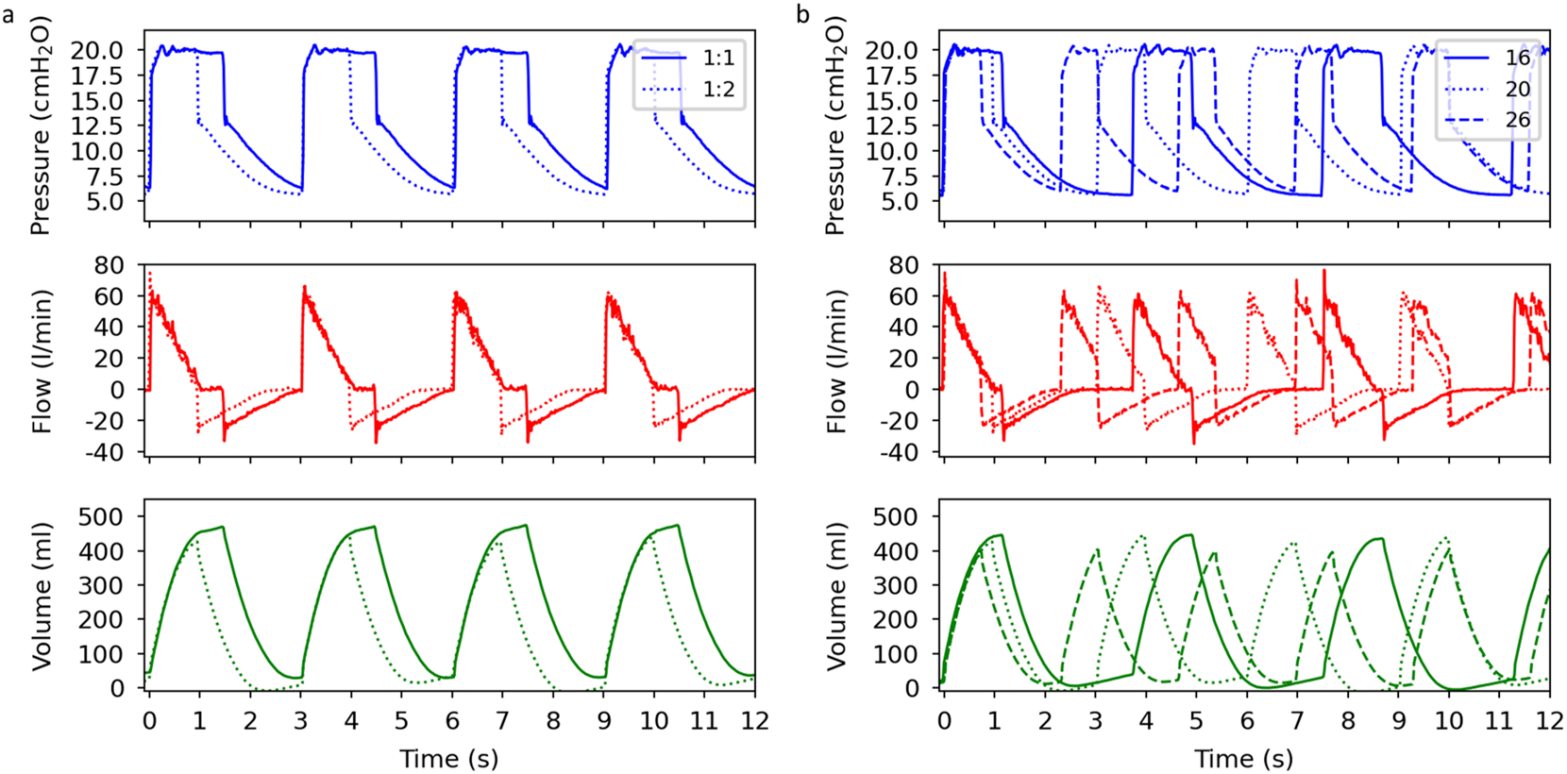
Plots of the airway pressure, flow, ventilation volume as a function of time. (**a**) Ventilation data plots acquired for the I:E ratios 1:1 and 1:2 while the respiratory rate and the P_IP_ value are set to 20 bpm and 20 cmH_2_O, respectively. (**b**) Ventilation data plots acquired for the respiratory rates 16 bpm, 20 bpm and 26 bpm while the I:E ratio is 1:2 and the P_IP_ value is 20 cmH_2_O. All measurements are conducted on an artificial lung and the pressure is generated with the CPAP machine u1 (see Table 3).

In our first test, we vary the I:E ratio while fixing the P_IP_ value and the respiratory rate. The acquired data for the airway pressure, flow, and volume as a function of time are presented in Fig. 2a. An evaluation of the inhalation and exhalation durations confirms the correct implementation of the I:E ratios with CARL. In the volume curve for the I:E ratio setting 1:1 (solid, green), we observe that the volume is always above zero (e.g. around 3 s) due to the short duration of the exhalation phase. This residual volume can lead to a hyperinflation of the patient’s lung, which must be reviewed by a trained staff member and can be beneficial depending on the patient’s condition^25,26^. Next, we vary the respiratory rate while keeping the P_IP_ value and the I:E ratio fixed. The ventilation curves are presented in Fig. 2b, where we observe an increase of the maximum ventilation volume as the respiratory rate decreases. This is expected since the duration of the inhalation phase decreases as the respiratory rate increases. We found that the respiratory rate agrees with the value that is set on CARL throughout the measurement.

Both tests also show that the plateau pressure and the PEEP do not depend on the respiratory rate and the I:E ratio. This supports our previous finding that CPAP machines can quickly adapt to the sudden pressure and flow changes induced by CARL. Similar to other ventilation solutions, both ventilation parameters can be easily implemented in the code for the internal computer^9–16^. As such, it should be possible to cover any range of values for the respiratory rate and the I:E ratio that are outlined in international standards^27,28^ or guidelines^20^.

### Patient-triggered/patient-cycled ventilation

**Figure 3:**
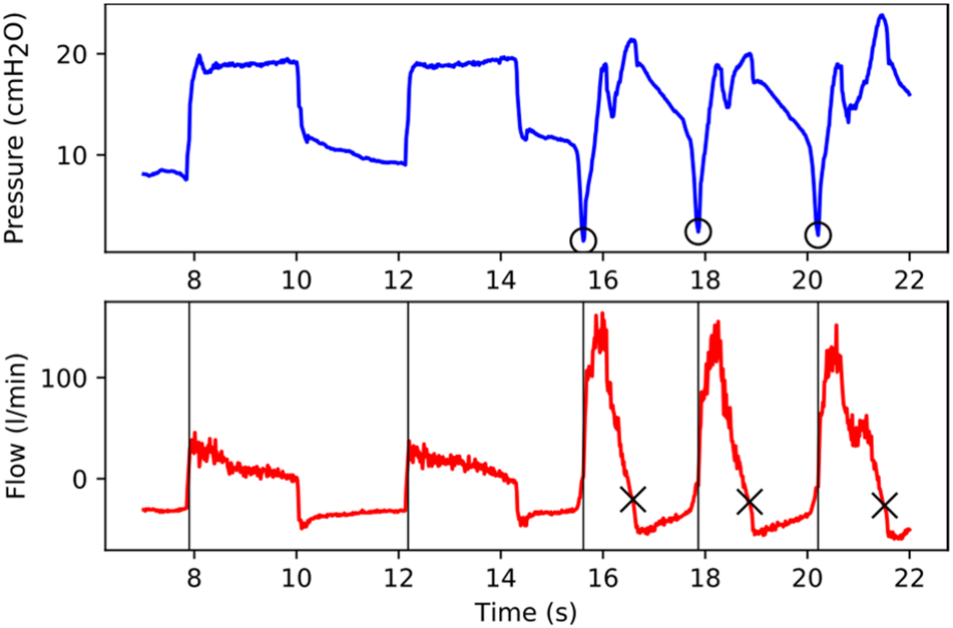
Illustration of patient-triggered and patient-cycled ventilation. The plots show the measured airway pressure (blue) and flow (red) as a function of time in the event of spontaneous breathing during ventilation (from ~ 16 s). The patient-initiated start of the inhalation and exhalation phase is marked as ‘o’ and ‘x’, respectively.

Patients who receive no sedation may breathe spontaneously during ventilation^29,30^. To support such events, the ventilator must be able to recognise the patient’s breathing effort, so that they can trigger and/or cycle the inspiration phase themselves. Our implementation relies on airflow monitoring which has also been implemented in another ventilation solution^12^. The ventilation curves in the case of spontaneous breathing during ventilation are presented in Fig. 3. Here, the start of the inhalation and exhalation phase is marked as ‘o’ and ‘x’, respectively. When no breathing effort is detected, e.g. when the patient’s breathing ceases, the ventilation is provided by the parameters set on CARL.

### Long-term testing

**Figure 4:**
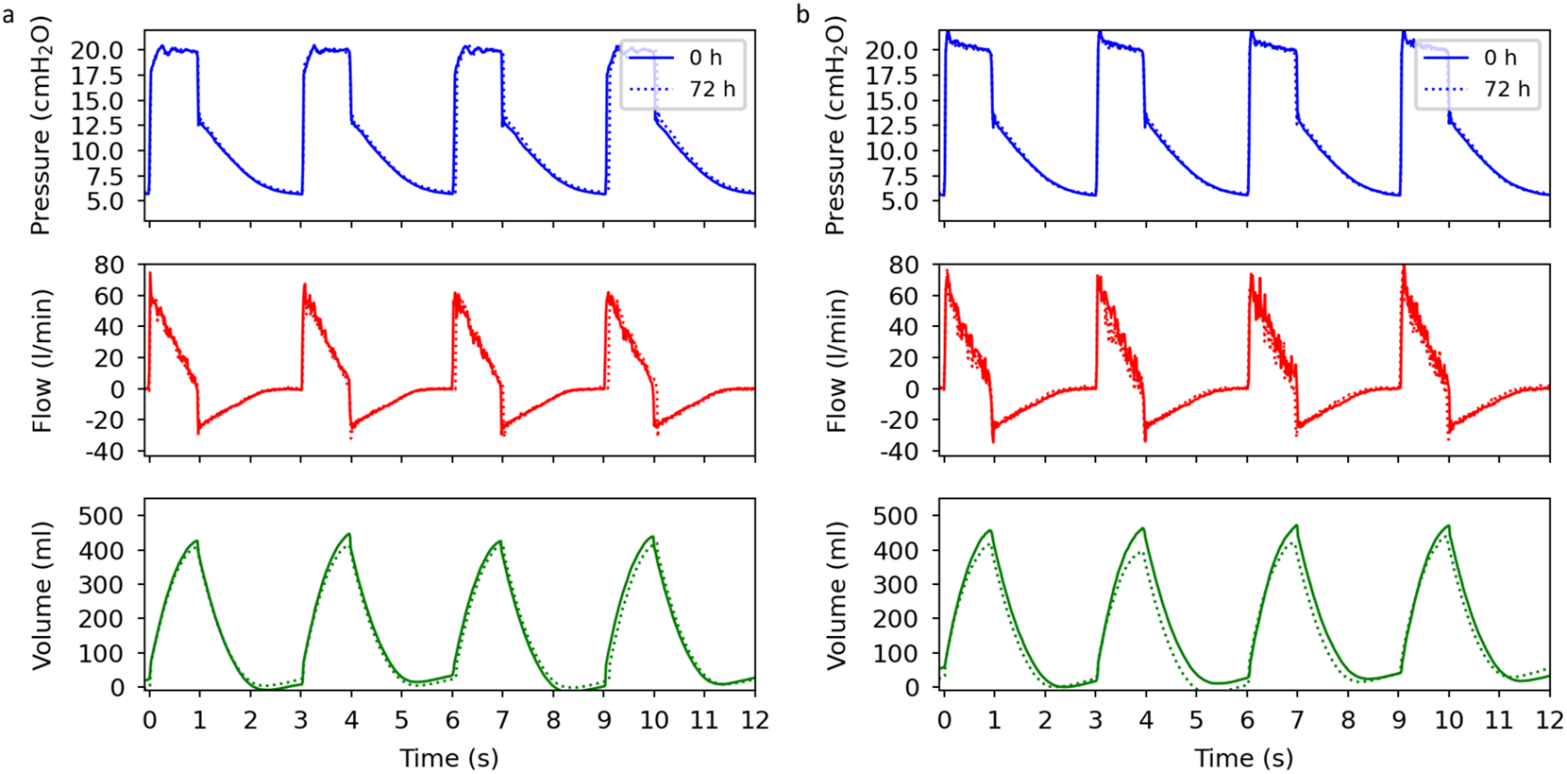
Plots of the airway pressure, flow, ventilation volume as a function of time for the CPAP machines (**a**) u1 and (**b**) u4 (see Table 3). The curves represent the ventilation performance after turning on the ventilator (0 h) and after running for 72 h without a break (72 h). All measurements are performed on an artificial lung. PIP: 20 cmH2O, Respiratory rate: 20 bpm, I:E ratio: 1:2.

Depending on the patient’s health condition, ventilators must operate for a few days to weeks straight^31,32^. CPAP machines, on the other hand, usually operate while the patient is sleeping. As such, these machines rarely run for more than a few hours per day. To assess if an extended operation period can have an impact on the ventilation performance, we chose two CPAP machines and measure the ventilation curves after 72 h of operation. During this period, we observed no shutdown of the CPAP machine. Figure 4a,b present the measurements for two CPAP machine models. For the machine in Fig. 4a, we see no changes in the ventilation performance after 72 h. In Fig. 4b, the positive flow reduces after 72 h, which results in a drop of the peak ventilation volume by 8%. On the other hand, both the respiratory rate and the I:E ratio remain unchanged for both ventilators. An evaluation of the baseline for the pressure and the flow curves reveals no shifts over the duration of operation.

Our long-term tests reveal that CARL can operate for an extended period, while the performance of the CPAP machine may drop after a few days. Therefore, it is important to constantly monitor the ventilation curves and, if necessary, replace the CPAP machine to match the patient’s needs.

## Discussion

The previous results demonstrate that our module CARL can repurpose CPAP machines to basic ventilators which provide ventilation pressures up to 20 cmH_2_O while maintaining a minimum PEEP. Such ventilators may be used on COVID-19 patients with mild symptoms since critically ill patients require higher pressures which common CPAP machines cannot reach^33^. To determine the suitable clinical environments, e.g. critical-care or emergency and transport, and applicable clinical cases, an extended testing phase as part of pre-/clinical trials and reviews against international standards or guidelines are required. A crucial part of these examinations are tests against different lung parameter values such as lung compliance and linear resistance. Here, we tested our ventilator on a lung with lung parameters in the range found in ISO 80601-2-12^27^. Thus, additional tests are required at a later stage to determine the suitable clinical environment for CARL-based ventilators.

To contribute to the patient’s safety and address accidental/unintentional adjustments on the ventilator a set of alarms is essential. In commercial medical-grade ventilators, the set of mandatory alarms depend on the targeted clinical environment and are outlined in international standards such as ISO/TR 21954^28^. Critical-care ventilators, for example, require additional alarms that monitor the airway pressure, e.g. PEEP or high pressure, the delivered volume and airway obstructions^27^. So far, CARL has acoustic and visual alarms that monitor the operation of the motor and the connection to the pressure sensors. Protections against high pressures or airway obstructions are currently carried out by a valve unit that is part of CARL’s design (see Sect. 6.2). Given that the pressure, flow, and volume are continuously read out in CARL, we can implement in our software all pressure, flow and volume related alarms that are required for the targeted clinical environment.

Since CARL-based ventilators are different to commercial medical-grade ventilators, additional training is necessary for the medical staff. For our ventilator solution, the medical staff must familiarise themselves with the CPAP machine and the CARL module. To reduce the training time required for CARL we use a clear and easy-to-use interface that allows constant patient monitoring and instant access to the ventilation settings, i.e. the respiratory rate and the two I:E ratios. While our basic ventilators require little time to set up and use, they should only be used by a trained staff member with ventilation expertise. This is because the performance of CPAP machines can vary with each model so that only constant monitoring and evaluation of the ventilation curves can lead to a successful patient treatment.

Owing to CARL’s modular design, we can develop additional components to include more functionalities without interfering with the ventilator design. One potential component is a module to supply oxygen to the patient at a given oxygen level. This will also imply the addition of an oxygenation measurement device.

Further adjustments to CARL’s design could be made to improve the infection control of the module. The design should allow the user to easily clean its surface within a reasonable time frame. In our case, this would primarily concern the components that are in the ventilation pathway. However, since these components are affordable and easy to produce, it may be safer to dispose and replace them instead. Owing to the working principle of CARL, our device could repurpose any continuous positive pressure machine to a basic ventilator. As such, CARL may be the starting point for an open-source ventilator solution. Such decisions, however, require testing the device carefully on animals or patients, which is beyond the scope of this report.

## Summary and outlook

The rapid dissemination of COVID-19 has led to a short-term shortage of ventilators worldwide. We believe that CPAP machines repurposed as stripped-down ventilators can help with the crisis in the short term. Our mass-producible solution could be used to vacate medical-grade ventilators for patients in critical conditions. However, in the worst case and after further development and regulatory approval, our solution may be used as a last resort to save the patient’s life.

It is important to note that CARL must be rigorously examined and tested before it can be used on humans. This includes stress tests for both hardware and software, performance measurements under clinical conditions and a biocompatibility evaluation of all materials in the ventilation pathway.

## Methods

### Ventilation specifications for a minimally acceptable ventilator

Our set of ventilation specifications is aimed at COVID-19 patients with mild symptoms as they may not require all functionalities provided by a medical-grade ventilator. A summary of the ventilation variables supported by our system is presented in Table 1. For the definition of the ventilation variables and modes, we use the terminologies and the guidelines provided by Charburn et al.^34^.

**Table 1:** Specifications for a minimally acceptable ventilator to treat COVID-19 patients with mild or moderate symptoms.

Ventilation variable	Description	Values
Triggering mechanism	A method to initiate the inspiration phase	Time-triggering, flow-triggering
Cycling mechanism	A method to end the inspiration phase	Time-cycling, flow-cycling
Breath control variable	A parameter that describes the mechanism to assist the patient’s breathing	Pressure-control (PC)
Breath sequence	A pattern of mandatory and/or spontaneous breaths	Intermittent mandatory ventilation (IMV)
Targeting Scheme	A method used by the ventilator to achieve a specific ventilation pattern	Set-point (s)
Ventilation Mode	A term to describe the set of ventilation operations based on the selected breath control variable, breath sequence and targeting scheme	PC-IMVs,s
I:E ratio	The ratio of the inspiration (I) and the expiration (E) phase during ventilation	1:1, 1:2
Respiratory rate	The number of breaths per minute (bpm). Here, it is the number of mandatory breaths per minute. The number of spontaneous breaths per minute cannot be lower than the number of mandatory breaths per minute	8–30 bpm
Positive end-expiratory pressure (PEEP)	The airway pressure at the end of the exhalation phase	At least 5 cmH_2_O
Peak inspiratory pressure	The airway pressure set at the CPAP machine	15–20 cmH_2_O
Maximum airway pressure	The upper-pressure limit in the patient’s airways	30 cmH_2_O

In addition to the ventilation specifications, we also implemented functionalities in CARL’s design that ensure the patient’s safety during therapy and the safe operation of CARL. These functionalities are summarised in Table 2.

**Table 2:** Design requirements for CARL to ensure the patient’s safety during ventilation.

Function	Description
Contamination protection	Protection of patient and medical equipment from pathogens
Constant monitoring (settings)	Visual display of airway pressure, tidal volume, and current ventilation settings
Constant monitoring (hardware and software)	Visual and acoustic signals for hardware and software failures during operation

### CARL

**Figure 5:**
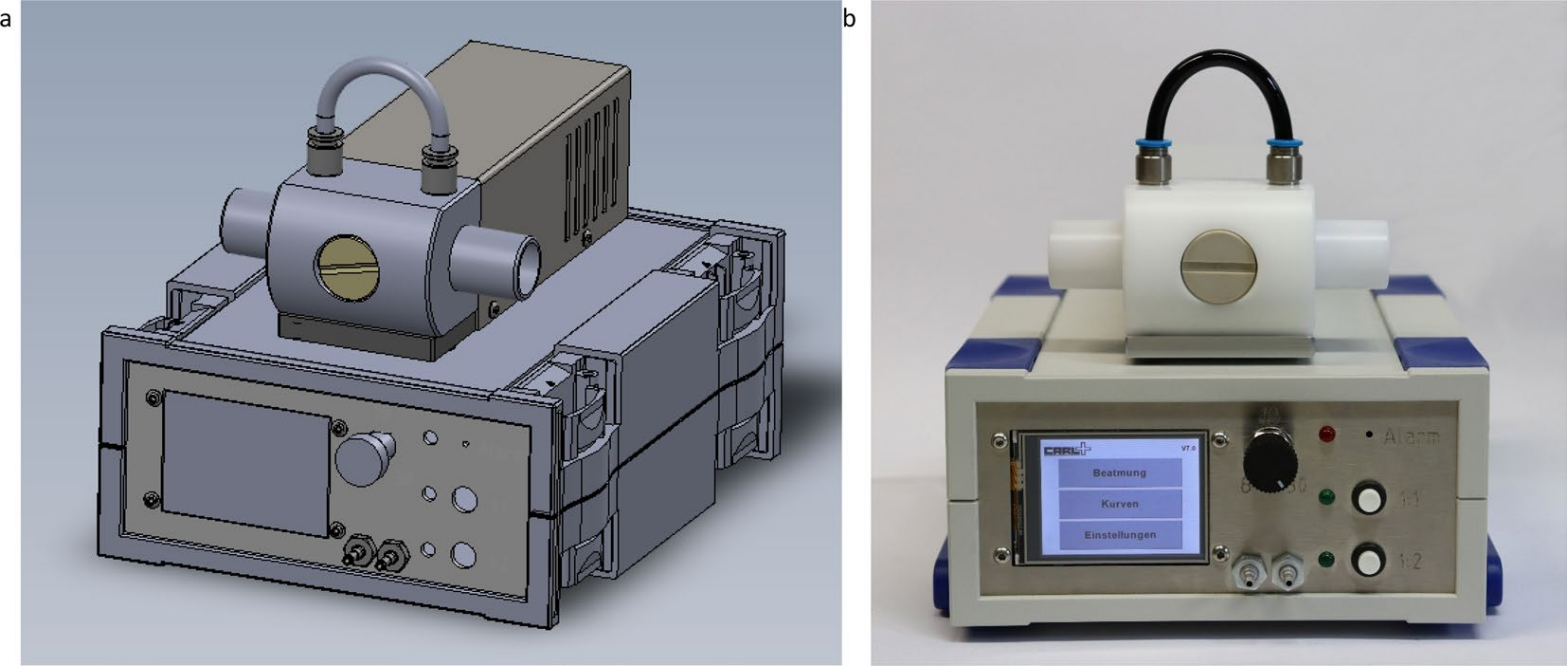
Illustrations of the module CARL. (**a**) 3D model of the module CARL. (**b**) Assembled model of CARL.

For the development of our module CARL, we aim for a mass-producible solution that can be manufactured worldwide. To fulfil this criterion, we developed a design that only uses easy-accessible hardware and electronic components. Our current design for CARL is presented in Fig. 5a,b, where the essential components are optimised for mass-production using plastic milling. We also developed and tested a version that can be built with a 3D printer. Subsequent tests show, however, that the results strongly depend on the 3D printer model and, thus, we focus on the plastic-milled version as it yields superior and more consistent results.

**Figure 6:**
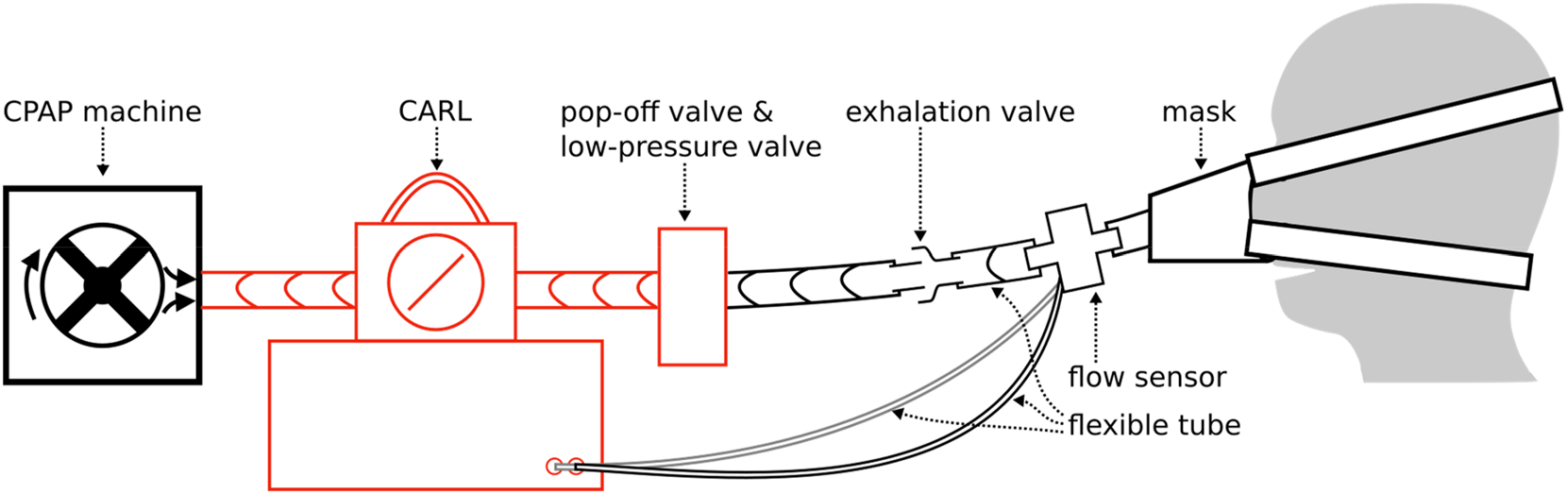
Schematic of a basic ventilator with a CPAP machine and the module CARL.

CARL serves as an intermediate module that we place in the ventilation pathway between the CPAP machine and the patient (see Fig. 6). The airway pressure that is generated by the CPAP machine in the ventilation pathway is regulated with a rotatable valve: when the valve is open, the airway pressure increases to the peak inspiratory pressure; when it is closed, the airflow stops and the airway pressure drops to zero. A flexible tube is used to bypass the valve and to allow for a continuous minimum airflow to the patient. That way, a minimum airway pressure can be maintained even when the valve is closed. The minimum pressure is determined by the diameter of the bypass channel. In the present case, it is adjusted to maintain a PEEP of at least 5 cmH_2_O at 20 cmH_2_O. To meet the set respiratory rate and I:E ratio during ventilation, the valve is attached to a computer-controlled motor. The patient receives air through a mask or an endotracheal/tracheostomy tube. Any excess air during the patient’s exhalation phase can escape through an exhalation valve that is placed between CARL and the patient.

We also designed a pop-off valve and a low-pressure valve to contribute to the patient’s safety. The former component makes sure that the airway pressure does not exceed 30 cmH_2_O, e.g. in the event of a cough. The low-pressure valve guarantees that patients can inhale spontaneously in the event of a hardware or software failure. Additional ventilation parameters, e.g. the FiO_2_ value, can be extracted by connecting a patient monitor to the provided connector.

To measure the airway pressure and the flow, we use a flow sensor (No. 281637, Hamilton Medical) that is connected to two implemented pressure sensors (AMS5915-0020-D-B and AMS5915-0050-D-B, Analog Microelectronics GmbH). The flow data is evaluated to determine the ventilation volume and to trigger and to cycle inspiration. As such, we can continuously monitor and evaluate the patient’s breathing behaviour during mechanical ventilation.

To comply with safety precautions while in operation, all electronic parts are protected by a professional casing. We use an Arduino Uno and an Arduino Nano to control all electric components in CARL. To implement the ventilation modes in Table 1, we use our own code that is written in C++ and only employs standard libraries. For all timing related functions, e.g. the respiratory rate, we use the standard Arduino function *millis()*, which returns the time that has passed since the program has been started^35^. After approximately 50 days, the time goes back (rolls over) to zero, which may cause timing issues during ventilation but is accounted for in our code.

### Interface/GUI

**Figure 7:**
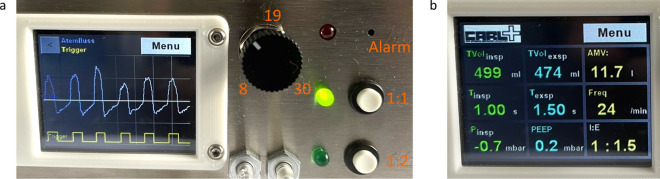
Photo of the user interface of CARL. (**a**) Entire interface with a display of a flow curve. Ventilation parameters are highlighted in orange. (**b**) Display of the current ventilation parameters and ventilation quantities.

The use of clear labels is essential for the safe use of CARL by the medical staff. To achieve this, all descriptions use standard terms that are recognised by qualified medical personnel. Figure 7a shows a photo of CARL’s user interface. To operate CARL, the user can set the desired I:E ratio and the respiratory rate. The red LED next to the description “Alarm” implies an error during operation. These errors can be defined and implemented into our code. On the display, the user can view the ventilation curves (Fig. 7a shows a flow curve) and the current ventilation parameters and ventilation quantities (Fig. 7b).

### Measurement of ventilation quantities

**Figure 8:**
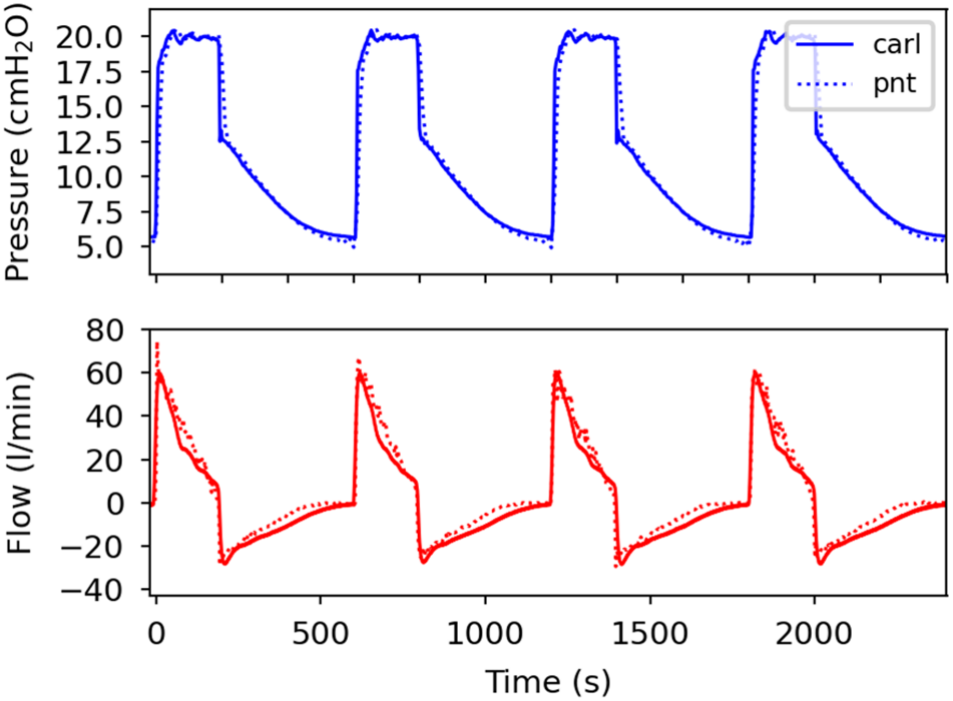
Comparison of measured pressure (blue) and flow (red) data acquired from an external calibrated system ( “pnt”) and CARL (“carl”). All measurements are conducted on an artificial lung and the pressure is generated with the CPAP machine u1 (see Table 3). P_IP_: 20 cmH2O Respiratory rate: 20 bpm, I:E ratio: 1:2.

CARL uses accessible pressure sensors to monitor the pressure and the flow that are different to those implemented in medical-grade ventilators. For the sensor used in this study, the accuracy at room temperature is reported as 1 cmH_2_O^36^. Since such sensors are mass-produced, their performance may vary with each product. Because of this, we must validate and calibrate all our measurements with an independent system. Here, we use a system that consists of a pneumotachometer (Model 3700A, Hans Rudolph Inc.) attached to a heater control (Model 3850, Hans Rudolph Inc.) and a DC bridge amplifier (MIO-0501, FMI GmbH). To acquire and evaluate the data from this setup we use the Embla RemLogic Software. For the calibration, we compare both data and adjust for systematic differences so that our data from the sensor matches the one from the external system. A representative result is presented in Fig. 8 which shows that both the pressure and the flow curves are in good agreement after calibration.

To release the excess gas during ventilation, we use an exhalation valve (Whisper Swivel II, Philips Respironics) with a microfilter attached to it. The filter reduces the concentration of released air contaminants in the environment and thus, the overall infection risk for the medical staff. As a patient model, we use an artificial lung (Test Lung 190, Maquet) that has a reported compliance and linear resistance value of 16 $\frac{\mathrm{ml}}{{\mathrm{cmH}}_{2}\mathrm{O}}$ and 20 $\frac{{\mathrm{cmH}}_{2}\mathrm{O}}{(\mathrm{l}/\mathrm{s})}$, respectively^37^.

In this study, we use five different CPAP machine models which are summarised in Table 3. We refer to them with the term in the column “Reference”.

**Table 3:** List of CPAP machine models.

Manufacturer	Model	Reference
Löwenstein Medical GmbH	Prisma SOFT	u1
Löwenstein Medical GmbH	Prisma 25 S	u2
Respironics Inc	REMstar C-Flex Int’l	u3
DeVilbiss healthcare	9055SE	u4
Löwenstein Medical GmbH	Phönix 3	u5
